# Plague in the Americas.

**DOI:** 10.3201/eid0707.017718

**Published:** 2001

**Authors:** A Ruiz

**Affiliations:** Pan American Health Organization, El Paso, Texas 79912, USA. ruizalfo@fep.paho.org


### Plague in the Americas

**Alfonso Ruiz**
Pan American Health Organization, El Paso, Texas, USA

Plague, caused by the bacteria *Y. pestis,* is a disease of rodents and their fleas that occasionally is transmitted to other animals and humans. Three worldwide pandemics, causing millions of deaths, have been recorded. The last one, which began in the 19th century, reached the Americas.

A few natural plague foci are found in the Americas: in the western United States; in northern Peru; in Chimborazo Province, Ecuador; and in the Department of La Paz, Bolivia. Several foci are also located in the semi-arid regions of northeastern Brazil. The population at risk in these areas is estimated at more than 16 million.

From 1994 through 1999, plague was reported in five American countries: Bolivia, Brazil, Ecuador, Peru, and the United States; approximately 1,700 cases were recorded, with 79 deaths (Table 1).

Several rodents have been identified as reservoirs. The main vector is the rodent flea *Xenopsylla cheopis*, although other species have been identified, particularly, in the United States (Table 2).

Three clinical forms of plague are recognized: bubonic, septicemic, and pneumonic. The septicemic and pneumonic forms are usually secondary to the bubonic form, and the bubonic form is the most common in the Americas. It is characterized by swelling of cervical, axillary, and inguinal lymph nodes, depending on the location of the portal of entry of the bacteria. The incubation period is from 3 to 6 days. Hematogenous dissemination of the bacteria to other organs and tissues may cause intravascular coagulation and endotoxic shock, producing dark discoloration in the extremities (so-called black death).

Laboratory confirmation of all forms is encouraged, either by microbiologic methods or serologic demonstration of antigens or antibody titers.

Infection mostly occurs through flea bites; however, infection can become airborne when a patient with pneumonic plague coughs. Humans are exposed to infection in the outdoor or household environment. Infections in the wild usually cause isolated or sporadic cases; this occurs in the United States and Brazil, where most infected persons are Indians, hunters, miners, and tourists.

Household infections occur when people, domestic animals (especially cats), and peridomestic rodents bring infected fleas into the house, exposing more persons. Raising guinea pigs inside homes, as they do in the Andean countries, is an additional risk factor for outbreaks. These animals become infected and multiply the infection by sharing their fleas with humans. Persons might also become infected through skin injuries when preparing the guinea pigs for cooking. Houses constructed with thatched walls and roofs or adobe walls are highly vulnerable to rodent activities (seen in plague-endemic areas of Andean countries). Improper storage of crops in patio areas or in the roof provides easy food access for rodents, facilitating transmission of plague.

Increased rainfall in a geographic area causes extensive changes to the surroundings, which can lead to the displacement of wild fauna, including rodents. The resulting soil moisture may improve crop production, or any other mammal food resources, and lead to an increase of plague hosts. These effects are difficult to register and correlate because plague events occur years later after the meteorologic phenomenon and ecologic modification. This has been the case with the El Nino-Southern oscillation phenomenon, which caused an unusual amount of precipitation in northern Peru. The ecology was modified over a wide area, resulting in the development of new crops, which then helped the rodent population increase.

In addition, deforestation to gain new lands for agriculture in areas known as natural foci of plague will eliminate most of the rodent predators and provide additional food and shelter to wild rodents, facilitating their rapid reproduction. Such was the case in the province of Chimborazo, Ecuador, where the inhabitants planted wheat crops after deforestation.

Epidemiologic characterization of areas where plague is prevalent, a potential risk, or silent will help establish surveillance and prevention measures. Obtaining serologic specimens from dogs is an effective tool for identifying areas

Table 1. Plague: Reported Cases and Deaths, 1994–1999

| | 1994 | | 1995 | | 1996 | | 1997 | | 1998 | | 1999 | |
|---|---|---|---|---|---|---|---|---|---|---|---|---|
| Country | Cases | Deaths | Cases | Deaths | Cases | Deaths | Cases | Deaths | Cases | Deaths | Cases | Deaths |
| Bolivia | 0 | 0 | 0 | 0 | 26 | 4 | 1 | 0 | 0 | 0 | 0 | 0 |
| Brazil | 0 | 0 | 0 | 0 | 1 | 0 | 0 | 0 | 0 | 0 | 0 | 0 |
| United States | 13 | 1 | 9 | 1 | 5 | 0 | 4 | 1 | 9 | 0 | 9 | 0 |
| Peru | 1,122 | 51 | 97 | 2 | 33 | 0 | 39 | 0 | 20 | 0 | 151 | 5 |
| Ecuador | 0 | 0 | 0 | 0 | 0 | 0 | 0 | 0 | *160 | 14 | 0 | 0 |
| Total | 1,135 | 52 | 105 | 3 | 55 | 4 | 44 | 1 | 189 | 14 | 160 | 5 |

*Estimate

Address for correspondence: Alfonso Ruiz, Pan American Health Organization, 5400 Suncrest Dr., Suite C-4, El Paso, TX 79912, USA; fax: 915-845-4361; e-mail: ruizalfo@fep.paho.org

Table 2. Plague in the Americas: Reservoirs and Vectors

| Country | Reservoirs | Vectors |
|---|---|---|
| Bolivia | *Akodon* sp | *Xenopsylla cheopis* |
| | *Rattus rattus* | *Pulex irritans* |
| Brazil | *Akodon* sp | *X. cheopis* |
| | *Oryzomys* sp | |
| | *Callomys* sp | |
| | *Bolomy* sp | |
| | *Monodelphis deomestica* | |
| Ecuador | *R rattus* | *P. irritans* |
| | *R. norvegicus* | |
| | *R. alexandrinus* | |
| | *Akodon mollis* | |
| | *Oryzomys* sp | |
| | *Phyllotis* sp | |
| | *Scirurus stramineus* | |
| United States | Marmot (*Cynomys* sp) | *Orchopeas sexdentatus* |
| | Rabbits | *Oropsylla montana* |
| | Rats (*Dipodomys* sp) | *Haplosyllus* sp |
| | Mice (*Peromyscus* sp) | *Diamanus* sp |
| | Terrestrial squirrel (*Citellus* sp) | *Thrasis* sp |
| Peru | *Akodon* sp | *X cheopis* |
| | *Oryzomys* sp | *Polygenes* sp |
| | *Sigmodon* sp | *Tiamastus* sp |
| | *Phyllotis* sp | *P. irritans* |
| | *R. rattus* | |
| | *Cavia porcellus* | |

where plague is prevalent because dogs are susceptible to *Y. pestis* infection. Although they rarely develop the disease, they can maintain detectable titers of antibodies for extended periods. Trapping rodents can also be used for surveillance to detect *Y. pestis* infection by microbiologic or serologic testing and for identifying the flea vectors.

Identifying and treating infected persons are priorities in plague-endemic areas. Streptomycin is the most effective antibiotic for treating plague. Tetracyclines are preferred for prophylactic use. Vaccination is not possible because no effective vaccines currently exist.

Education is appropriate in the areas where infection is known and where people are at risk. Messages can be delivered that take into account the local, cultural, and ethnic characteristics of the communities.

Flea surveillance and control with proper insecticides could be carried out by a local community. Periodic application of insecticides inside and outside homes is important in reducing the flea population in infected areas. Other prevention measures could be implemented on the basis of local risk assessments; for example, in Peru when improper storage of grains attracted rodents inside the houses, small silos were designed to store the goods.

We recognize that plague is still in the Americas and human population is rapidly growing. New lands are being used for new settlements, and new crops are being grown for food production. Many species of rodents can serve as reservoirs, not only for *Y. pestis* infection but also for other emerging infections, and at any moment a new outbreak might appear. Local, cross-cutting, and interdisciplinary approaches are encouraged to implement adequate surveillance of rodentborne diseases.

### Intercontinental Transmission of West Nile Virus by Migrating White Storks

**Mertyn Malkinson,* Caroline Banet,* Yoram Weisman,* Shimon Pokamonski,† Roni King,‡ and Vincent Deubel§**

*Kimron Veterinary Institute, Beit Dagan, Israel; †Israeli Veterinary Services, Beit Dagan, Israel; ‡Nature Reserves Authority, Israel; and §Pasteur Institute, Paris, France

In September and October 1998, West Nile (WN) virus was isolated from a flock of 1,200 migrating white storks (*Ciconia ciconia*) that had landed in Eilat, a town in southern Israel. Inclement weather conditions of strong, hot westerly winds had forced them to fly under considerable physical stress to reach Eilat. The storks were fledgelings, less than 1 year old, that had hatched in Europe. Analysis of blood samples taken from several birds within days of their arrival showed the presence of WN virus–neutralizing antibodies. Sequence analysis of the envelope glycoprotein gene of the stork isolate showed almost complete identity with a sample isolated from a dead goose in Israel in 1998.

Because this Eilat flock was migrating southward for the first time and had not previously flown over Israel, we assume that it became infected with WN virus in Europe. The presence of virus-neutralizing antibodies in stork serum samples collected from German flocks provided additional evidence that the birds contracted WN virus in Europe. These findings indicate that the recent epizootic of WN virus in Israeli geese had its origin in Europe, where the virus had been circulating in epidemic proportions since 1996. Epidemiologic studies of eastern European epidemics indicate that WN virus may now be endemic in southern Europe.

Address for correspondence: Mertyn Malkinson, Kimron Veterinary Institute, Beit Dagan, P.O. Box 12, Israel 50250; fax: 972-3-9681739; e-mail: martinm@moag.gov.il

